# Comparison of exercise electrocardiography and stress perfusion CMR for the detection of coronary artery disease in women

**DOI:** 10.1186/1532-429X-14-36

**Published:** 2012-06-14

**Authors:** Simon Greulich, Oliver Bruder, Michele Parker, Julia Schumm, Stefan Grün, Steffen Schneider, Igor Klem, Udo Sechtem, Heiko Mahrholdt

**Affiliations:** 1 Robert-Bosch-Medical Center, Auerbachstrasse 110, 70376, Stuttgart, Germany; 2 Contilia Heart and Vascular Center, Elisabeth Hospital Essen, Essen, Germany; 3 Duke University Medical Center, Durham, NC, USA

## Abstract

**Background:**

Exercise electrocardiography (ECG) is frequently used in the work-up of patients with suspected coronary artery disease (CAD), however the accuracy is reduced in women. Cardiovascular magnetic resonance (CMR) stress testing can accurately diagnose CAD in women. To date, a direct comparison of CMR to ECG has not been performed.

**Methods and results:**

We prospectively enrolled 88 consecutive women with chest pain or other symptoms suggestive of CAD. Patients underwent a comprehensive clinical evaluation, exercise ECG, a CMR stress test including perfusion and infarct imaging, and x-ray coronary angiography (CA) within 24 hours. CAD was defined as stenosis ≥70% on quantitative analysis of CA.

Exercise ECG, CMR and CA was completed in 68 females (age 66.4 ± 8.8 years, number of CAD risk factors 3.5 ± 1.4). The prevalence of CAD on CA was 29%. The Duke treadmill score (DTS) in the entire group was −3.0 ± 5.4 and was similar in those with and without CAD (−4.5 ± 5.8 and −2.4 ± 5.1; P = 0.12). Sensitivity, specificity and accuracy for CAD diagnosis was higher for CMR compared with exercise ECG (sensitivities 85% and 50%, P = 0.02, specificities 94% and 73%, P = 0.01, and accuracies 91% and 66%, P = 0.0007, respectively). Even after applying the DTS the accuracy of CMR was higher compared to exercise ECG (area under ROC curve 0.94 ± 0.03 vs 0.56 ± 0.07; P = 0.0001).

**Conclusions:**

In women with intermediate-to-high risk for CAD who are able to exercise and have interpretable resting ECG, CMR stress perfusion imaging has higher accuracy for the detection of relevant obstruction of the epicardial coronaries when directly compared to exercise ECG.

## Background

Coronary artery disease (CAD) is the leading cause of morbidity and mortality in women [1]. The assessment of CAD in women is challenging compared with men for several reasons. The clinical presentation is often with atypical symptoms and the predictive power of traditional cardiac risk factors is different in women compared to men [2]. Based on assessment of symptoms and risk factors, the majority of women being evaluated for chest pain syndromes have an intermediate pre-test probability of CAD. In this group of patients accurate noninvasive tests are an indispensable component in the diagnostic work-up [3]. However, well-established noninvasive tests for the diagnosis of CAD all have substantial limitations in women in predicting significant angiographic CAD [4]. Furthermore, the prevalence of CAD in women presenting with chronic anginal pain as well as acute coronary syndromes is lower compared with men [5,6]. Thus, based on Bayesian principles the predictive value of noninvasive tests is reduced [7]. Additionally, the estimation of sensitivities and specificities of noninvasive tests based on reported results is frequently limited by post-test referral bias in which only women with abnormal test results are referred to the reference test, resulting in enhanced diagnostic sensitivity and diminished specificity [8].

Noninvasive diagnostic testing with exercise electrocardiography (ECG) is the oldest, least costly, and most commonly used form of stress testing. This test appears to be less accurate in women for the diagnosis of CAD, and both lower sensitivities and specificities have been reported compared to men [9,10]. This gender difference remains even when combining the interpretation of ST-segment deviation with exercise time and exercise induced symptoms into the Duke Treadmill Score (DTS) [11,12]. These difficulties posed on the clinical determination of CAD probability have led to speculation that stress imaging approaches may be an efficient initial alternative to exercise ECG in women [13], however few data are available to support this approach.

Stress perfusion CMR has been shown previously to accurately diagnose CAD in the clinical setting in a mixed gender population [14] as well as in women [15]. The aim of the present study was to compare exercise ECG (ST-segment deviation alone) and the DTS with CMR stress testing for the detection of CAD in women with invasive coronary angiography as the gold standard.

## Methods

### Study population

Women with chest pain or other signs and symptoms suggestive of CAD, who were referred for elective coronary angiography (CA) were screened for study enrollment. Patients were contacted by telephone the day before admission for scheduled angiography, and the first patient meeting study criteria who agreed to participate was recruited. The exclusion criteria were patients with known CAD including those with prior myocardial infarction (MI) or revascularization procedures, as well as contraindications to MRI (e.g. pacemaker) or adenosine (e.g. high-grade AV-Block). Institutional Review Board approval was received and written informed consent was obtained from all enrolled patients. All patients underwent x-ray angiography, exercise ECG, and CMR within 24 hours. Some of the patients were also included in another study [15]. However, the comparison between stress CMR and exercise ECG is reported for the first time for all patients included.

### Protocol

On the day of study enrollment a complete medical history including responses to a Rose chest pain questionnaire was obtained. Blood samples were drawn after an overnight fast for glucose, lipid profile, and high-sensitivity C-reactive protein. CAD risk factors were defined using Framingham heart study definitions [16]. A 12-lead electrocardiogram was registered and scored for Q-waves and bundle-branch block using Minnesota codes [17].

### Exercise electrocardiography

All patients underwent symptom-limited cycle ergometer testing with continuous 12-lead ECG monitoring. A 25-Watt incremental protocol every 2 minutes was used and a 12-lead ECG hard copy was recorded before exercise and at the end of each exercise stage (every 2 minutes), at peak exercise and at 2-minute intervals during recovery. Patient symptoms, rest and peak heart rate, blood pressure, and any ECG changes were noted. The test was discontinued for limiting symptoms (angina, dyspnea, fatigue), abnormalities of rhythm or blood pressure, or marked ST-segment deviation (>0.2 mV in the presence of typical angina), or attainment of age-predicted maximal heart rate (calculated as 220 – age) [12].

All exercise ECG recordings were interpreted by consensus of two experienced readers. The ECG criterion for a positive test was greater than or equal to 1 mm of horizontal or downsloping ST-segment deviation (depression or elevation) in any lead except aVR for at least 60 to 80 milliseconds (ms) after the end of the QRS complex, either during or after exercise. Patients with left-bundle branch block on resting ECG, which interferes with interpretation of the exercise test, were considered non-diagnostic and were not included in the final analysis [12].

The exercise capacity in metabolic equivalents (METs) was estimated based on patients body weight and maximum achieved level of exercise on the cycle ergometer in Watts [18]. The Duke Treadmill Score (DTS) was calculated based on the METs, the amount of ST-segment deviation, and exercise angina index [12,19]. The largest net ST-deviation, either elevation or depression in any lead except avR was entered into the formula, if ST-segment deviation was less than 1 mm, the value entered was 0. Treadmill angina was graded based on the following scale: 0 = no angina during exercise, 1 = nonlimiting angina during exercise, and 2 = exercise-limiting angina. The typically observed range for the DTS is −25 (highest risk) to +15 (lowest risk), and patients were dichotomized into low risk (DTS score ≥5), moderate risk (DTS score 4 to −10), and high risk (DTS score ≤ −11) groups as previously proposed [11].

### Cardiovascular magnetic resonance

Details of the CMR scan protocol and analysis have been reported previously [14]. In brief, steady-state free-precession cine images for assessment of LV function were acquired in multiple short-axis (every cm throughout the LV) and 3 long-axis views. Adenosine (140 μg·kg^-1^·min^-1^) gadolinium (0.07 mmol/kg gadodiamide, GE Healthcare, Buckinghamshire, United Kingdom) first-pass imaging for assessment of stress perfusion was then performed using a saturation-recovery, single-shot, gradient-echo sequence (90˚-prepulse before each slice; TE, 1.1 ms; delay time, 85–100 ms; temporal resolution, 110–125 ms; voxel size, 3.1 x 1.8-2.5 x 8 mm; iPAT factor 2) as previously described. Repeated first-pass images without adenosine 15 minutes later were performed for assessment of rest perfusion. Five minutes after rest perfusion (additional 0.07 mmol/kg gadodiamide), Late Gadolinium Enhancement (LGE) imaging was performed using a segmented inversion-recovery technique in the identical views as cine-CMR. The image acquisition protocol was completed in about 45 minutes. A 1.5-T scanner (Siemens Sonata, Erlangen, Germany) with a phased-array receiver coil was used.

Scans were analyzed by consensus of experienced observers from Stuttgart (S.G.), Duke (I.K.) and Essen (O.B.) who were blinded to patient identity, clinical information, exercise ECG and the angiography results. In cases consensus could not be achieved an additional senior observer (H.M.), also blinded to all patient information, made the final decision. Regional parameters were assessed using a 17-segment model as previously described for cine and LGE [14,20]. A perfusion defect was defined as a regional dark area, that 1) persisted for >2 beats while other regions enhanced during the first-pass of contrast through the LV myocardium, and 2) involved the subendocardium. Stress and rest images were assessed using 16 segments (segment-17 at apex was not visualized) and each segment was scored using a 4-point scale: 0, normal; 1, probably normal; 2, probably abnormal; 3, definitely abnormal. This scoring system was chosen to allow dichotomization of results into normal (≤1) and abnormal (≥2) at the same time provide a range of scores for ROC curve analysis. Dark rim artifact was not regarded as perfusion deficit using previously described criteria [21].

Standard methods were used to quantify left ventricular volumes (enddiastolic and endsystolic), ejection fraction, and mass using Argus software (Siemens, Erlangen, Germany) on short axis Cine images [22].

### Coronary angiography and analysis by coronary artery territory

X-ray CA was performed by standard techniques and analyzed masked to identity, clinical information, exercise ECG and CMR results. In patients with stenosis >40% determined visually by the consensus of two experienced cardiologists, computer-assisted quantification of luminal diameter stenosis (QCA) was performed (except for subtotal stenosis). Significant CAD was defined as ≥70% narrowing of the luminal diameter in at least one projection of at least one major epicardial artery, or ≥50% narrowing of the left main [3]. Additionally, we evaluated detection of at least intermediate grade stenoses by applying a cutoff of ≥50%.

### Statistical analysis

Continuous data are expressed as mean ± standard deviation. Comparisons between groups were made using two sample *t* tests for continuous data and *χ*^2^ tests for discrete data. The Fisher exact test was used when the assumptions of the *χ*^2^ test were not met. The McNemar test was used to compare sensitivities, specificities and the diagnostic accuracy of CMR and exercise ECG. The receiver-operator characteristic (ROC) curve analyses was performed to compare the diagnostic performance of the CMR and exercise ECG. Statistical tests were 2-tailed; P < 0.05 was considered statistically significant.

## Results

### Study population

**Figure 1:**
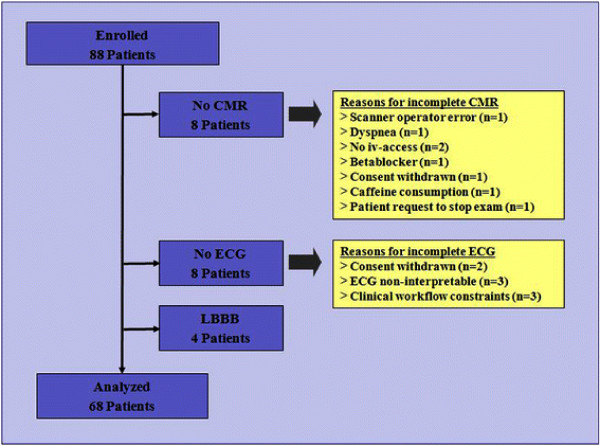
**Outline of Patient Enrollment.** CMR = Cardiovascular Magnetic Resonance, ECG = exercise electrocardiography, LBBB = left bundle-branch block.

A total of 88 women were enrolled in the study from September 2004 to December 2005 (Figure 1). Eight women did not have a complete CMR test. In one case scanner operator error lead to incomplete data, one patient requested to stop the scan due to discomfort in the scanner. In 6 cases, imaging was omitted because of non-CMR related issues: one had xanthines in the morning before the scan; one had beta blockers within 12 hours before the scan; one withdrew consent; in two, intravenous access could not be obtained; and in one, there was severe adenosine-induced dyspnea which led to early termination of the protocol. The woman with dyspnea, which quickly resolved after stopping adenosine, had the only adverse event during stress-testing. In twelve women the exercise ECG could not be completed or was non-diagnostic: four women had left bundle-branch block, two refused exercising and withdrew consent from study participation, in three the ECG documentation was of inadequate quality for interpretation, three women were sent to cardiac catheterization before the research exercise test could be performed due to clinical workflow constraints. The remaining 68 patients, who underwent both an exercise ECG and stress CMR were included in the final analysis.

Table 1 summarizes the patient characteristics. The majority (61%) had angina by Rose chest pain questionnaire, however the proportion of women with angina was similar in the groups with and without obstructive CAD (p = 0.45). Patients had several CAD risk factors (3.5 ± 1.4), but the prevalence of CAD risk factors was similar in women with CAD and those without CAD. The only differences noted were older age and higher prevalence of hyperlipidemia in females with CAD. Only 2 women were pre-menopausal, all others were post-menopausal. The mean body mass index was 27.3 ± 4.6 kg/m^2^ in the entire group, and was similar in both women with (27.2 ± 3.6 kg/m^2^) and without CAD (27.4 ± 5.0 kg/m^2^; P = 0.88). The majority (79%) had an abnormal noninvasive stress-test as part of their clinical workup prior to elective CA, in 14 patients (21%) symptoms alone were considered highly suggestive of CAD by the treating physician and CA was deemed indicated without a preceding noninvasive test.

**Table 1:** Baseline Characteristics

**Characteristic**	**Entire Group(n=68)**	CAD *(n=20)	**No CAD(n=48)**	**P**
**Age** (yrs)	66.4±8.8	70.8±5.9	64.5±9.2	**0.001**
***CAD risk factors***				
Diabetes	16 (24%)	5 (25%)	11 (23%)	0.85
Hypertension	45 (66%)	16 (80%)	29 (60%)	0.12
Cigarette smoking	21 (30%)	5 (25%)	16 (33%)	0.50
Hyperlipidemia	41 (60%)	18 (90%)	23 (48%)	**0.001**
Family history of CAD	33 (49%)	9 (45%)	24 (50%)	0.71
*Menopause*	66 (97%)	20 (100%)	46 (96%)	0.35
*Obesity (BMI ≥ 30 kg/m*^*2*^*)*	19 (28%)	6 (30%)	13 (27%)	0.81
Number of risk factors	3.5±1.4	4.0±1.2	3.4±1.5	0.13
**Rose chest pain questionnaire**				
Angina	41 (61%)	13 (68%)	28 (58%)	0.45
**Medications**				
Statins	20 (29%)	8 (40%)	12 (25%)	0.22
Beta-blockers	35 (51%)	14 (70%)	21 (44%)	**0.05**
Aspirin	43 (63%)	15 (75%)	28 (58%)	0.19
ACE-inhibitors	27 (40%)	8 (40%)	19 (40%)	0.97
Nitrates	7 (10%)	2 (10%)	5 (10%)	1.00††
Diuretics	19 (28%)	3 (15%)	16 (33%)	0.12
Hormone replacement†	32 (48%)	8 (40%)	24 (52%)	0.41
**Blood tests** ‡				
Fasting glucose (mg/dL)	99.3±16.6	101.7±15.6	98.4±17.1	0.52
Lipids				
Total cholesterol (mg/dL)	225.3±36.8	237.6±33.8	220.2±37.2	0.08
LDL (mg/dL)	137.0±32.5	147.3±28.5	132.7±33.4	0.09
HDL (mg/dL)	59.8±16.8	59.9±19.6	59.7±15.6	0.97
Triglycerides (mg/dL)	146.7±70.0	152.3±71.1	144.4±70.2	0.68
hs-CRP (mg/dL)	0.36±0.5	0.48±0.7	0.32±0.4	0.35
**Indication for angiography**				
Positive stress nuclear study	9 (13%)	5 (25%)	4 (8%)	
Positive stress echo study	6 (9%)	3 (15%)	3 (6%)	
Positive treadmill ECG study	39 (57%)	9 (45%)	30 (63%)	
Clinical symptoms alone	14 (21%)	3 (15%)	11 (23%)	
**12-lead ECG**				
*Q-wave****||***	5 (7%)	1 (5%)	4 (8%)	1.00††
*RBBB****#***	2 (3%)	1 (5%)	1 (2%)	1.00††

CAD = coronary artery disease, BMI = body mass index, ACE-inhibitor = angiotensin-converting-enzyme inhibitor, LDL = low density lipoprotein, HDL = high density lipoprotein, hs-CRP = high-sensitivity C-reactive protein, RBBB = right bundle branch block.

* CAD defined by x-ray coronary angiography (see text).

† Current or ever-users (peri- or postmenopausal women).

‡ Blood tests were acquired within 24 hours of CMR in 54 patients for fasting glucose (non-diabetic patients only).

|| Minnesota codes 1-1-1 to 1-2-7.

# Minnesota codes 7-2-1 and 7-2-2.

†† Fisher exact test (two-tailed).

### Exercise ECG

The exercise ECG test results are shown in Table 2. There was no significant difference in heart-rate and blood-pressure response in women with or without CAD. Overall, the exercise duration was 5.4 ± 1.9 min and maximum achieved level of exercise capacity was 6.0 ± 1.5 METs. Women with CAD did achieve similar exercise times and METs as women without CAD. The occurrence of exercise-induced angina was similar among women with and without CAD. Among the 16 women who developed angina during exercise (both exercise limiting and non-limiting angina), 8 (50%) had CAD on CA. Conversely, among the fifty women without angina during exercise 11 (22%) had CAD (P = 0.08). In two patients, symptoms were not documented. Target heart rate (THR), calculated as ≥85% of age-predicted maximal heart rate, was reached in 49 (72%) of studies. The percentage of patients who reached THR was similar in women with and without CAD. In women who reached the THR the sensitivity, specificity, and accuracy were 43%, 74%, and 65% for detection of CAD (≥70%), whereas in women not reaching THR the sensitivity, specificity, and accuracy were 67%, 69%, and 68%, respectively. Of the 19 women not reaching THR, 7 patients developed angina (4 of which had CAD) and the remaining 12 women developed either fatigue, dyspnea, or other non-anginal symptoms during exercise. The DTS was −3.0 ± 5.4 in the entire cohort, and was similar in the women with and without CAD (−4.5 ± 5.8 vs. -2.4 ± 5.1; p = 0.12). The majority of women (n = 56) were in the moderate risk category by the DTS, 16 (29%) of these were diagnosed with CAD on CA. Four (6%) women each were in the low-risk and high-risk groups by the DTS. CAD was present in 1 woman in the low-risk group and in 2 women in the high-risk group.

**Table 2:** Exercise ECG Test Results

**Parameter**	**Entire Group(n=68)**	**CAD†(n=20)**	**No CAD(n=48)**	**P**
**Rest HR (beats/min)**	76.9±13.4	72.5±11.9	78.7±13.7	0.08
**Rest SBP (mmHg)**	127.0±20.6	128.5±18.6	126.4±21.5	0.71
**Rest DBP (mmHg)**	82.7±11.6	85.8±12.9	81.3±10.9	0.15
**Peak HR (beats/min)**	136.7±19.1	134.4±18.3	137.7±19.5	0.52
**Peak SBP (mmHg)**	188.0±28.7	185.4±16.8	189.1±32.6	0.54
**Peak DBP (mmHg)**	88.9±16.4	91.7±13.2	87.7±17.6	0.37
**Exercise duration (min)**	5.4±1.9	5.3±1.6	5.4±2.1	0.76
**THR reached**	49 (72%)	14 (70%)	35 (73%)	0.81
**METs**	6.0±1.5	5.8±1.1	6.0±1.7	0.62
**DTS**	−3.0±5.4	−4.5±5.8	−2.4±5.1	0.12

ECG = electrocardiogram, CAD = coronary artery disease, HR = heart rate, SBP = systolic blood pressure, DBP = diastolic blood pressure, THR = 85% of age-predicted maximum heart rate, METs = metabolic equivalents, DTS = Duke Treadmill Score,

† CAD defined by x-ray coronary angiography (see text).

### Stress CMR

The infusion duration for adenosine was 3.2 ± 0.5 minutes. The heart-rate was 73.2 ± 12.0 beats/min at rest and 97.6 ± 14.4 beats/min at peak stress. The majority (82%) experienced symptoms during adenosine infusion; most frequent symptoms were dyspnea and chest discomfort described as pain, pressure, tightness or burning, followed by head pressure, flush, nausea, and neck discomfort. Among the 68 women who completed imaging, five had frequent ventricular ectopy and one had atrial fibrillation chronically and during imaging. All 68 were considered to have evaluable images and were included in the analysis. Table 3 shows the results of quantitative analysis of cardiac volumes and function. Overall, left ventricular ejection fraction was preserved (65.9 ± 10.1%) with normal cardiac volumes.

**Table 3:** Quantitative Cine CMR Results

**Parameter**	**Entire Group** **(n=68)**	**CAD**† **(n=20)**	**No CAD** **(n=48)**	**P**
**LV Ejection Fraction (%)**	65.9±10.1	63.8±10.1	66.8±10.0	0.28
**LV-EDV (ml)**	95.4±22.8	86.7±18.2	99.1±23.7	**0.04**
**LV-ESV (ml)**	33.4±15.8	32.0±13.6	34.0±16.7	0.64
**LV-mass (g)**	103.6±22.5	103.5±25.5	103.6±21.4	0.99
**LV-EDV Index (ml/m**^**2**^**)**	53.6±12.1	50.2±9.5	55.0±12.9	0.14
**LV-ESV Index (ml/m**^**2**^**)**	18.8±9.4	18.4±7.4	19.0±10.2	0.83
**LV-mass Index (gm/m**^**2**^**)**	58.3±12.4	60.0±14.4	57.5±11.6	0.45

CMR = cardiovascular magnetic resonance, CAD = coronary artery disease, LV = left ventricle, EDV = end-diastolic volume, ESV = end-systolic volume.

† CAD defined by x-ray coronary angiography (see text).

### Comparison between exercise ECG and CMR

**Figure 2:**
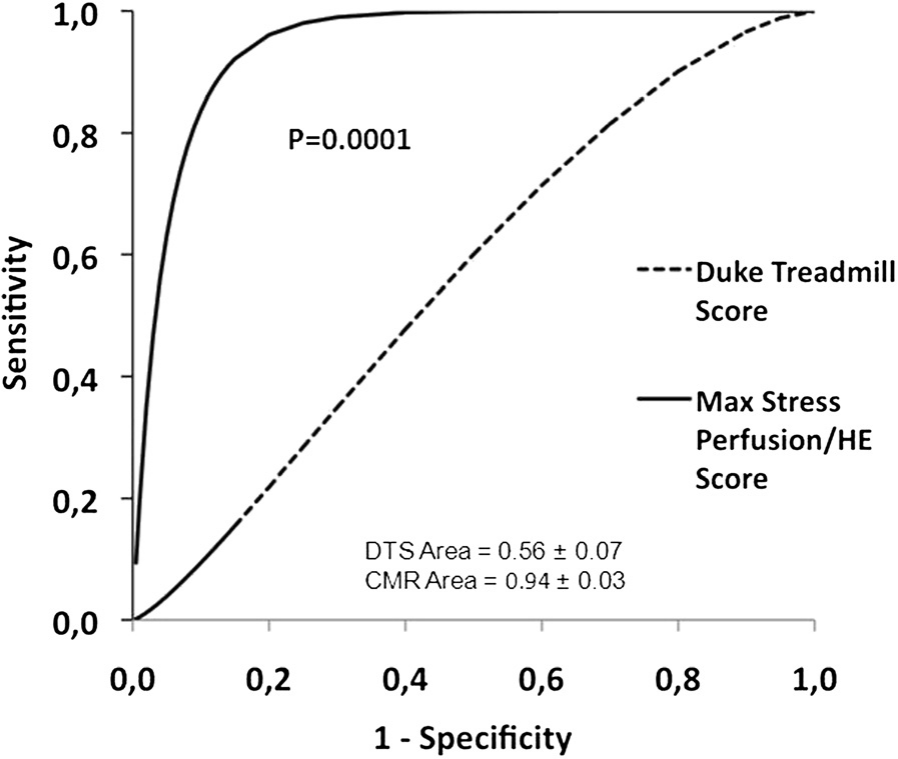
**Receiver-Operator Characteristic Curves.** Receiver-operator characteristic curve analyses comparing CMR and the Duke Treadmill Score for the detection CAD in all patients. The diagnostic accuracy of CMR was significantly greater than the Exercise ECG even when considering the Duke Treadmill Score (P = 0.0001). AUC = area under the curve.

**Figure 3:**
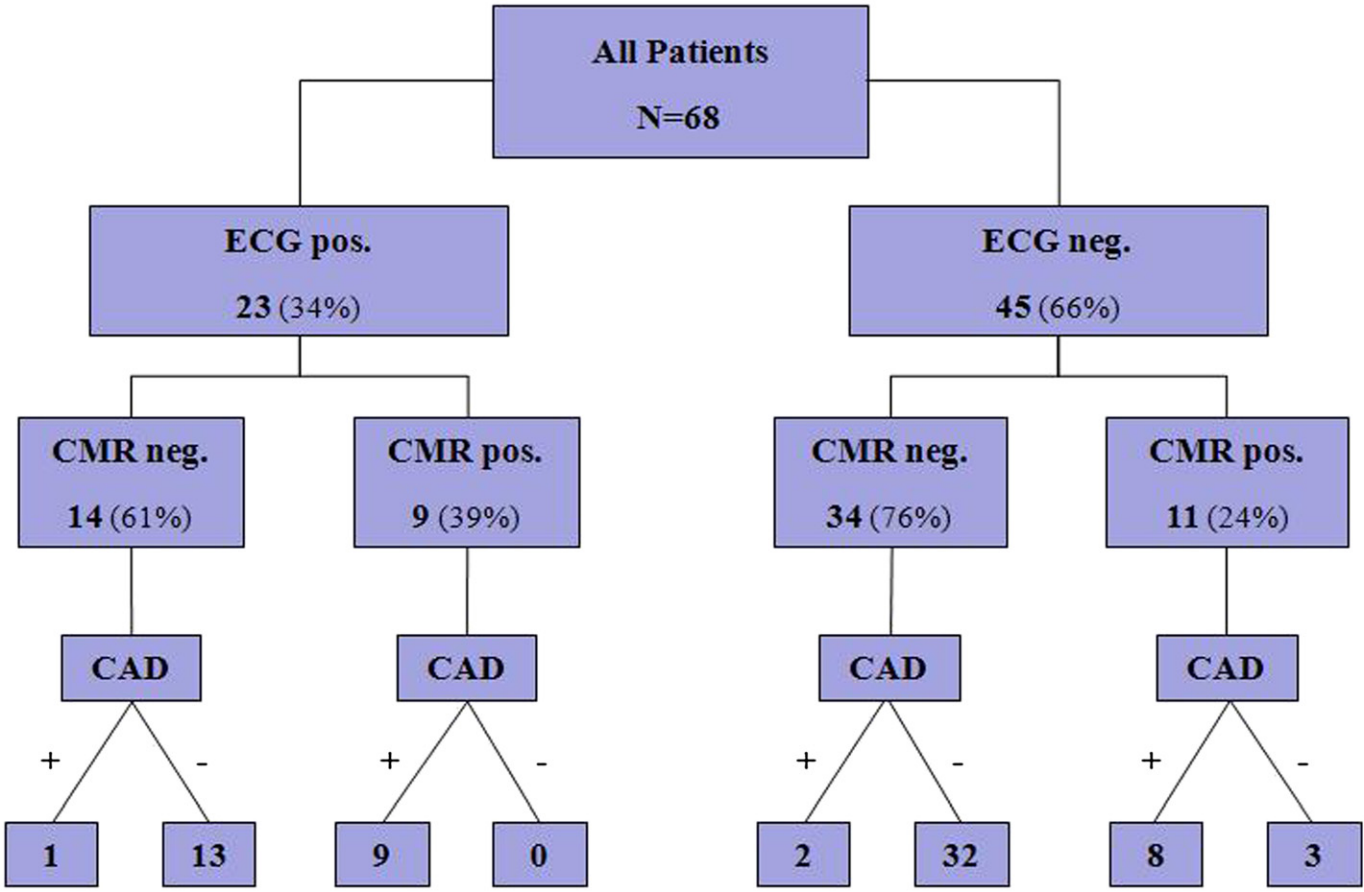
**Combined Outcome of Exercise ECG and CMR For the Detection of CAD.** ECG = Exercise Electrocardiogram, CMR = Cardiovascular Magnetic Resonance, CAD = Coronary Artery Disease.

CAD (≥70%) on CA was found in 20 (29%) women, and 26 (38%) women had stenosis ≥50%. The diagnostic performance of CMR and exercise ECG according to CAD severity is shown in Tables 4 and 5. CMR stress testing (including LGE, according to the diagnostic algorithm proposed by Klem at al [14].) had higher sensitivity (85% vs 50%; P = 0.02), higher specificity (94% vs 73%; P = 0.01), and higher diagnostic accuracy (91% vs 66%; P = 0.0007) compared with exercise ECG. Eight women had multi-vessel disease on CA, CMR detected all eight women and exercise ECG detected four women. Even after considering clinical information (exercise capacity and symptoms) in addition to ST-segment response in the DTS, the accuracy of CMR was higher compared to exercise ECG (area under ROC curve 0.94 ± 0.03 vs 0.56 ± 0.07; P = 0.0001, Figure 2). The combined results of CMR and exercise ECG in all patients are shown in Figure 3. Overall, CMR and exercise ECG were concordant in 43 (63%) patients and discordant in 25 patients. Among the patients with discordant results, 14 had a positive exercise ECG and a negative CMR. In 13 (93%) of those patients no obstructive CAD was found on CA. The remaining patient (1/14 = 7%) had a mid LAD stenosis of 60-70% by visual analysis, which was graded 72% by QCA. In this patient no intervention was performed and no adverse event occurred during more than 3 years of follow-up. Conversely, 11 patients had a negative exercise ECG and a positive CMR. In 8 (73%) of those patients, significant CAD was found on CA.

**Table 4:** Diagnostic Performance of CMR Stress Testing for the Detection of CAD According to Disease Severity

**CMR Stress Test**		
	CAD ≥ 70%*	**CAD ≥ 50%**
**Sensitivity**	85% (17/20)	65% (17/26)
**Specificity**	94% (45/48)	93% (39/42)
**Diagnostic Accuracy**	91% (62/68)	82% (55/68)
**PPV**	85% (17/20)	85% (17/20)
**NPV**	94% (45/48)	81% (39/48)

CMR = Cardiovascular Magnetic Resonance, CAD = Coronary Artery Disease,

PPV = Positive Predictive Value, NPV = Negative Predictive Value.

* or ≥50% left main disease.

**Table 5:** Diagnostic Performance of Exercise ECG for the Detection of CAD According to Disease Severity

**Exercise ECG**		
	CAD ≥ 70%*	**CAD ≥ 50%**
**Sensitivity**	50% (10/20)	50% (13/26)
**Specificity**	73% (35/48)	76% (32/42)
**Diagnostic Accuracy**	66% (45/68)	66% (45/68)
**PPV**	44% (10/23)	57% (13/23)
**NPV**	78% (35/45)	71% (32/45)

CMR = Cardiovascular Magnetic Resonance, ECG = Electrocardiogram.

CAD = Coronary Artery Disease, PPV = Positive Predictive Value.

NPV = Negative Predictive Value.

* or ≥50% left main disease.

## Discussion

The major finding of the present study was that perfusion CMR has a higher accuracy for the detection of CAD when directly compared to exercise ECG in women who are capable of maximal exercise and have an interpretable resting ECG. The sensitivity of 85% and specificity of 94% with CMR was obtained in symptomatic women with intermediate-to-high risk for CAD. We minimized pre-test referral bias by excluding patients with known CAD (or prior MI) and normal studies. Post-test referral bias was minimized in that all patients underwent the reference test (coronary angiography) independent of the results of both exercise ECG and CMR. Both noninvasive tests were performed for research purposes only, and did not affect patient management.

Exercise ECG is considered the initial test of choice in women with suspected CAD [3], which is based on a large number of studies demonstrating its utility for the detection of CAD. In a meta-analysis including 19 exercise ECG studies with 3721 women the mean sensitivity was 61%, and mean specificity was 70%. However, a wide range of sensitivities (27%-91%) and specificities (46-86%) was observed in the individual studies, which is largely attributed to differences in prevalence of CAD (ranging from 18% to 75%), different influence by post-test referral bias, and different thresholds for interpreting a test as positive [10]. The sensitivity and specificity of exercise ECG in our study were 50% and 73%, which is comparable to previous reports in over 1600 women undergoing exercise ECG with a sensitivity of 47% and specificity of 73% [23].

Stress perfusion CMR is a relatively new noninvasive method with high diagnostic accuracy for the diagnosis of CAD, which has been demonstrated in various patient populations [24]. Few studies have assessed the utility of CMR stress testing specifically in women [15,25-27]. The prior reports vary in imaging technology (older pulse sequences and different coil technology) [25], pre-test probability (patients with known CAD and prior MI included) [26], and CAD prevalence (low-risk ED patients with chest pain) [27]. The sensitivities and specificities in the previous studies ranged from 57%-95% and 75%-100%, which concurs with our results. None of the prior reports however, directly compared the diagnostic performance of CMR to exercise ECG in the same patients.

Exercise ECG is the oldest noninvasive test for evaluation of patients with chest pain, it is simple, widely available, relatively inexpensive, and there is substantial experience with this test. However, this test is considered less accurate in women compared with men. Reduced sensitivity has been attributed to lower prevalence of CAD and the inability of many women to exercise to maximum aerobic capacity [9,28,29]. Exercise ECG is generally considered also as less specific in women than in men even after correction for post-test referral bias [30]. Among the non-Bayesian factors, syndrome X, differences in microvascular function, and possibly hormonal differences have been discussed [12,31]. Current guidelines recognize the limitations in accuracy of exercise ECG and that stress-imaging approaches in general may be an efficient initial alternative in women. However, presently available data concerning direct comparison of exercise ECG to stress imaging tests in the same patient is insufficient to justify routine stress imaging as the initial test for CAD in women [12]. CMR has emerged as an attractive new noninvasive test with an improved spatial resolution compared to nuclear techniques without the use of ionizing radiation. However, availability is limited to expert centers and the cost compared to exercise ECG is relatively high. The present study is a first step to investigate comparative effectiveness between exercise ECG and CMR stress testing by directly comparing results of both studies in the same populations.

Limitations of the present study are that not all potential sources of pretest referral bias were removed because patients were selected from those already scheduled for invasive angiography. In addition, invasive CA is not necessarily the ideal gold-standard for comparison as functional significance of coronary obstruction and luminal diameter stenosis are moderately correlated. Furthermore it is important to keep in mind that the algorithm used for CMR analysis [14] is intended to detect significant obstruction of the epicardial coronaries compared to invasive CA (e.g. >70% stenosis). Thus, perfusion defects that were regarded as artifacts according to the algorithm used in the present analysis may in fact represent microvascular dysfunction. Since microvascular dysfunction is hardly detectable by conventional invasive CA these patients were regarded as “healthy” by CA, and correctly identified as such by CMR explaining the high specificity, despite the possible presence of microvascular dysfunction responsible for the clinical complaints of these women. Therefore, additional data with regard to the role of microvascular dysfunction is needed to fully understand the diagnostic performance of CMR in the setting of women with chest pain or other signs and symptoms suggestive of CAD.

The present study did also not include a comprehensive cost-comparison. A lower cost of the exercise ECG test however, does not necessarily translate into lower overall cost of patient care, because the sum of the cost of additional downstream testing and interventions may be higher when the initial exercise ECG is less accurate than CMR imaging test [32]. Cost analysis poses several challenges. The cost of a false positive ECG results is readily quantifiable by the cost of unnecessary cardiac catheterization. However, the cost of a false negative test is more difficult to quantify as it involves not only cost associated with morbidity (hospitalization, procedures) but also cost related to mortality, which is much more difficult to quantify.

## Conclusion

The present study demonstrates that in women with intermediate-to-high risk for CAD who are able to exercise and have interpretable resting ECG, CMR stress testing has higher accuracy for the detection of relevant obstruction of the epicardial coronaries. These findings warrant further investigations to evaluate both accuracy as well as cost to justify CMR stress testing as the initial test in this population.

## Competing interests

The authors declare that they have no competing interest.

## Authors’ contributions

SG contributed to the idea and design of the study, recruited the patients, acquired and analyzed the data, and wrote the report. OB, MP, JS, SG, and SS contributed to the idea and design of the study, analysis of the data, and revision of the report. IK and US contributed to the idea and design of the study, acquisition and analysis of the data, and revision of the report. HM designed the study, contributed to the acquisition and analysis of the data, and wrote the report. All authors read and approved the final manuscript.
